# Obituary: Dr John MG Davis PhD, DSc, FRCPath

**DOI:** 10.1186/1743-8977-8-34

**Published:** 2011-12-21

**Authors:** Anthony Seaton

**Affiliations:** 1 Emeritus Professor of Environmental and Occupational Medicine The University of Aberdeen, King's College, Aberdeen, AB24 3FX, UK; 2 8 Avon Grove, Edinburgh EH4 6RF, UK

## 

John Davis, who died aged 76 on October 21^st ^2011, was a leading researcher into asbestos and coal related diseases. In his role as head of pathology at the Institute of Occupational Medicine (IOM) in Edinburgh he led a group of scientists who were to make important contributions not just to the understanding of classical occupational diseases but also to the recent literature on nanotoxicology.

John was born in Sussex, England, on March 25^th^, 1935, and retained an apt and lifelong interest in cricket and nature, the latter leading to a childhood enthusiasm for science. From the local grammar school he went to Caius College Cambridge on a scholarship to study medicine but he was diverted to zoology, leading an expedition to the Gabon in 1961 to study bird behaviour, his recording of their song being used by the naturalist and broadcaster, David Attenborough. He became interested in electron microscopy and wrote his PhD on the ultrastructural signs of x-radiation of rat hepatocytes. He then obtained an industrial fellowship in the Cambridge University Department of Pathology and became assistant director of research, developing his interest in asbestos and its effects on the lung. This was of course the time when the malignant disease mesothelioma was described in relation to asbestos exposure in South Africa by Chris Wagner and his colleagues, and fears were beginning to be expressed that what was then a very rare tumour could become more common; sadly this proved to be the case. The asbestos industry had set up an organisation to research these issues and decided to base its research effort in the IOM, John moving there in 1971 to lead this research as head of the pathology group.

In Edinburgh John's researches were divided between coal miners' diseases and asbestos/synthetic vitreous fibres. The work on coal, in collaboration with David Lamb, Anne Ruckley and others, led to the key finding of a relationship between coal dust exposure and emphysema, a crucial piece of evidence that assisted in the recognition of dust exposure as a risk factor for chronic obstructive lung disease in miners, and thus compensable in UK regulation. For decades this had been a controversial area; John's group's work helped settle this to the benefit of many miners. It also contributed, its primary purpose, towards a scientific justification of tighter standards to prevent lung disease in miners.

While the coal research was in an area of controversy, research into asbestos was even more so. There is a rather inglorious history in occupational medicine of major industries sponsoring research with strings attached, and it has been stated that the asbestos industry was guilty of this. When I arrived as Director at the Institute in 1977 I did indeed find that the contract allowed the industry's representatives to have a veto on publication, though they had never exercised it. I was able quickly to convince them that this was not in their best interest and it was removed from the contract. No work carried out by John and his colleagues was ever prevented from publication, though the industry did not agree to fund research in epidemiology when we tried to broaden our programme.

The work of John's group was concerned mainly with the reasons that asbestos fibres cause the various known diseases, asbestosis, pleural fibrosis and mesothelioma. He collaborated with physicists in understanding the relationships between fibre dimensions and toxicity and, particularly importantly, the relevance of fibre solubility in tissue. This work assisted in framing the paradigm of fibre pathogenicity, emphasising length, diameter and durability over the chemical characteristics of the fibre. The most important outcome of this research was the development of an approach to predicting the toxicity of new fibres, many produced as a substitute for asbestos in its many earlier applications in industry. Under John's leadership, the focus of the research changed from asbestos to these synthetic vitreous fibre substitutes as the use of the former mineral declined.

John's last important role at the IOM came in 1988 when the owner, British Coal decided to close it down as a cost saving measure. He and I embarked on a mission to raise funds in order to set it up as a self-funding charitable research foundation, and he was successful in persuading the Colt Foundation and the companies involved in producing non-asbestos fibres to fund a £1 million programme of research into the safety of such fibres, using the asbestos research as a model. John sat on the steering committee of this research programme after formally retiring in 1990 from the about- to- be- independent IOM.

John was a man of wide culture and interests. He was also a notably modest and rather shy person, and a scientist of the highest integrity. His work was recognised by his DSc and election to Fellowship of the Royal College of Pathologists, a singular honour for a non-medically qualified pathologist at the time. Most importantly, he had the ability to excite his young colleagues in research and to let them have their heads. Among these was Ken Donaldson who started with him as a laboratory technician and became professor of respiratory toxicology at Edinburgh University, a leading researcher into nanotoxicology, work derived directly from his early studies with John. No fewer than ten other young scientists working with him over two decades obtained PhDs, the majority having started as technicians.

John had married Julia in 1961 and they had two daughters. Sadly, although he remained active in retirement his last decade was one of increasing and ultimately very severe disablement from Parkinson's disease. Characteristically, one of his last acts was to donate his nervous system for research into this disease.

Prof Anthony Seaton

Edinburgh, Scotland

a.seaton@abdn.ac.uk

4th November 2011

